# Helical Majorana fermions in $d_{x^{2}-y^{2}}$ + *id_xy_*-wave topological superconductivity of doped correlated quantum spin Hall insulators

**DOI:** 10.1038/srep24102

**Published:** 2016-04-11

**Authors:** Shih-Jye Sun, Chung-Hou Chung, Yung-Yeh Chang, Wei-Feng Tsai, Fu-Chun Zhang

**Affiliations:** 1 Department of Applied Physics, National University of Kaohsiung, Kaohsiung, Taiwan, R.O.C.; 2 Electrophysics Department, National Chiao-Tung University, HsinChu, Taiwan, 300, R.O.C.; 3 Physics Division, National Center for Theoretical Sciences, HsinChu, Taiwan, 300 R.O.C.; 4 Department of Physics, National Sun Yat-Sen University, Kaohsiung, Taiwan, R.O.C.; 5 Department of Physics, Zhejiang University, Hangzhou, China; 6 Collaborative Innovation Center of Advanced Microstructures, Nanjing, China

## Abstract

There has been growing interest in searching for exotic self-conjugate, charge-neutral low-energy fermionic quasi-particles, known as Majorana fermions (MFs) in solid state systems. Their signatures have been proposed and potentially observed at edges of topological superconcuctors with non-trivial topological invariant in the bulk electronic band structure. Much effort have been focused on realizing MFs in odd-parity superconductors made of strong spin-orbit coupled materials in proximity to conventional superconductors. In this paper, we propose a novel mechanism for realizing MFs in 2D spin-singlet topological superconducting state induced by doping a correlated quantum spin Hall (Kane-Mele) insulator. Via a renormalized mean-field approach, the system is found to exhibits time-reversal symmetry (TRS) breaking 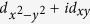-wave (chiral *d*–wave) superconductivity near half-filling in the limit of large on-site repulsion. Surprisingly, however, at large spin-orbit coupling, the system undergoes a topological phase transition and enter into a new topological phase protected by a pseudo-spin Chern number, which can be viewed as a persistent extension of the quantum spin Hall phase upon doping. From bulk-edge correspondence, this phase is featured by the presence of two pairs of counter-propagating helical Majorana modes per edge, instead of two chiral propagating edge modes in the *d* + *id*′ superconductors.

Searching for topological states of quantum matters constitutes one of the central and fundamental issues in condensed matter systems. The growing interest in topological insulators (TIs), which support gapless edge (or surface) states protected by time-reversal symmetry (TRS) while the bulk remains insulating^1^,^2^, is one prime example. Of particular interest are topological superconductors which support gapless self-conjugate, charge-neutral fermionic quasi-particle excitations^3^. These excitations which reflect non-trivial topological bulk properties are localized at the edges, known as Majorana fermions (MFs).

Much effort has been put in searching for signatures of Majorana fermions in solid state materials. One-dimensional semiconductor nano-wires with strong spin-orbit (SO) coupling under a magnetic field proximity to a s-wave superconductor have been proposed theoretically to host MF at both ends of the wire^4^,^5^,^6^, and also studied experimentally^7^,^8^,^9^,^10^,^11^. Similar ideas have been proposed in 2D systems where chiral MFs exist at the edges of spin-triplet, *p*–wave (odd-parity) superconductors^12^,^13^,^14^.

While realization of the above systems relies on TRS breaking by the Zeeman field, time-reversal invariant topological superconductors (TRITOPs)^15^,^16^,^17^,^18^ have recently been proposed to host two time-reversal pairs of helical MFs at edges in repulsively interacting SO coupled nano-wire proximity to either a s-wave^6^,^16^ or a *d*–wave^19^ superconductor at each end of the wire. Proposals to realize TRITOPs in 2D systems include the spin-triplet *p*_*x*_ ± *ip*_*y*_ superconductors^15^, the bi-layer Rashba system^20^, and in exciton condensates^21^.

In this paper, we suggest a novel mechanism for realizing *helical* Majorana fermions in 2D spin-singlet *chiral* superconductors with TRS-breaking pairing gap–by directly doping correlated 2D quantum spin Hall insulators (QSHIs or 2D TIs) on honeycomb lattice^22^,^23^. A paradigmatic model for QSHIs is the Kane-Mele (KM) model^23^,^24^, which shows a non-trivial *Z*_2_ topological (or spin Chern) number and supports helical edge states protected by TRS. The half-filled KM model with strong electron correlations is in the Mott-insulating (MI) phase^25^, while superconductivity appears upon doping. Attractive candidates to realize correlated QSHIs on honeycomb lattice include: graphene with enhanced Kane-Mele SO coupling (∼20 *meV*) by doping with heavy adatoms^26^, *In*_3_*Cu*_2_*VO*_9_^27^,^28^, *β*–*Cu*_2_*V*_2_*O*_7_^28^,^29^, and Iridium-based honeycomb compounds *X*_2_*IrO*_3_(*X* = *Na* or *Li*) with strong SO coupling and electron correlations^25^,^30^,^31^,^32^.

Via renormalized mean-field theory (RMFT) approach^33^,^34^, we find the spin-singlet TRS breaking 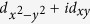-wave superconductivity to appear at the ground state where the chiral edge states have been shown to occur^35^. Surprisingly, for sufficiently large SO coupling compared with the superconducting gap, instead of chiral edge states, we find gapless helical MFs to appear at each ribbon edge. This seemingly un-expected feature comes as a result of persistent extension of the quantum spin Hall phase with non-trivial pseudo-spin Chern number upon doping. A novel pseudo-spin Chern to chiral topological quantum phase transition is identified.

## Results

### Model Hamiltonian

**Figure 1:**
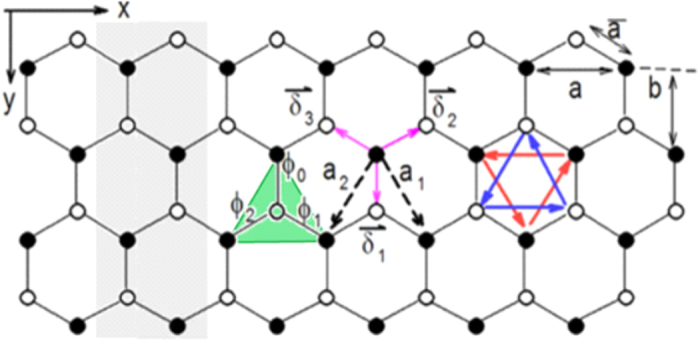
Honeycomb lattice of a finite-sized zigzag ribbon of the tight-binding Kane-Mele t-J model with the ribbon size *N* = 8 being twice the number of zigzag chains along *x*-axis. Nearest-neighbor and next-nearest-neighbor lattice vectors are 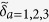, **a**_*i* = 1,2_ with an unit length of 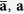, respectively. We set **a** = 1 here. The gray shaded region represents for the super-unit-cell of the zigzag ribbon, which repeats itself along *x*-axis. The filled (open) dots stand for the sites on sub-lattice *A*(*B*). The three phases for *d* + *id*′ pairing gap are defined (see shaded green triangle) as: *ϕ*_0_ = 0, *ϕ*_1(2)_ = −(+)2*π*/3.

The Hamiltonian of the Kane-Mele t-J (KM-tJ) model is given by^23^:


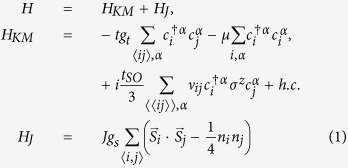


where α = ↑, ↓ stands for the spin index, 〈*i*, *j*〉 and 〈〈*i*, *j*〉〉 refer to the nearest-neighbor (NN) and next-nearest-neighbor (NNN) sites, respectively (see Fig. 1). Here, *v*_*ij*_ = 1 for *i*, *j* ∈ *A* and *v*_*ij*_ = −1 for *i*, *j* ∈ *B* in the SO coupling; 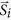 refers to the electron spin operator on site *i*, defined as: 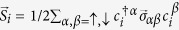, 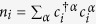 is the electron density operator, and the anti-ferromagnetic spin-exchange couping 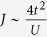 can be derived via the second-order perturbation from the Kane-Mele Hubbard model in the limit of a strong on-site Coulomb repulsion *U* ≫ *t*. Due to breaking of the *SU*(2) symmetry of the Kane-Mele Hubbard model at half-filling, a small effective ferromagnetic spin-exchange coupling *J*′ ≪ *J* term between NNN sites is generated via SO coupling^25^, which is neglected here (The *J*′ term favors the magnetic order in *XY*–plane^25^ and may induce spin-triplet superconductivity upon doping). We also drop the Rashba SO coupling for simplicity.

The *H*_*J*_ term has been known to favor the spin-singlet pairing. To address superconductivity of the model, we apply RMFT based on Gutzwiller projected single-occupancy constraint due to the proximity of the Mott insulating ground states, known to describe the ground state of *d*–wave cuprate superconductors in qualitative agreement with those via variational Monte Carlo approach^33^,^34^.

The spin-exchange *J* term within RMFT reads (see the methods section):


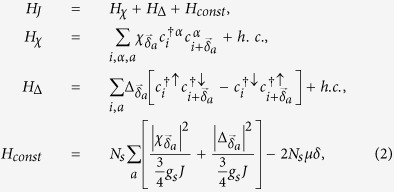


where *a* = 1, 2, 3, *N*_*S*_ is the total number of sites, 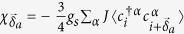, 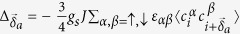, and 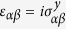. Here, 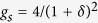 (see the Methods section). Based on the *C*_6*v*_ symmetry of the underlying lattice, the pairing symmetry of 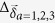 may take the following forms^36^,^37^: (i) extended *s*–wave: 
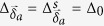
, (ii) 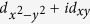
–wave (denoted also as *d* + *id*′): 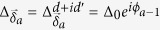 with *ϕ*_0_ = 0, *ϕ*_1(2)_ = −(+)2*π*/3 (see Fig. 1)^38^. The Fourier transformed pairing gap Δ_*k*_ for a periodic 2D lattice reads: 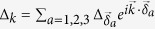.

The mean-field Hamiltonian 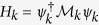 on a periodic lattice in the basis of 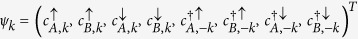 is given by the 8 × 8 matrix 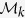:


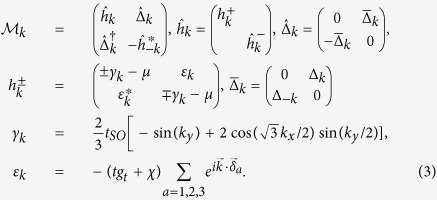


with *g*_*t*_ = 2*δ*/(1 + *δ*) (see the Methods section). The Hamiltonian Eq. (3) possesses both the Particle-hole (PH) symmetry: 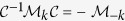, 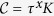 (with *τ*^*x*^ being the Pauli matrix on particle-hole space and *K* being complex conjugation) as well as sub-lattice symmetry: 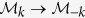 for *c*_*A*,*k*_ → *c*_*B*,*k*_^37^. The matrix 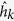, describing the KM model, shows TRS: 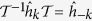 where 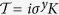 is the time-reversal operator taking 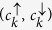 to 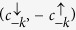. However, 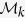 breaks the TRS for *d* + *id*′ superconducting order parameter: 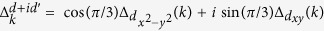 with 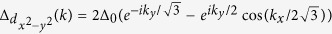, 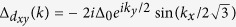, and 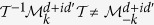. The mean-field free energy reads: 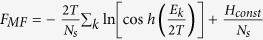 with *E*_*k*_ > 0 being positive eigenvalues and *N*_*s*_ the number of sites. We diagonalized the mean-field Hamiltonian *H*_*k*_ on a finite-sized zigzag ribbon with *N*_*s*_ = *N*/2 zigzag chains and *N* = 56 is set as the total number of sites along *y*–axis throughout the paper.

### Bulk and edge properties

The mean-field variables are solved self-consistently by minimizing the free energy both for a periodic lattice and a finite-sized ribbon (see supplementary materials). Compared to the TRS extended *s*–wave, we find 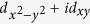
–wave pairing is the ground state^38^. Same pairing symmetry has been reported in superconducting phase of the doped graphene in the absence of the spin-orbit coupling^35^,^37^,^39^,^40^,^41^, which was argued to support two co-propagating chiral edge states at low energies with a non-trivial topological winding number *N*_*TKNN*_ = ±2^42^. The superconducting transition temperature *T*_*c*_ is estimated as *T*_*c*_ ∼ *g*_*t*_Δ_0_.

**Figure 2:**
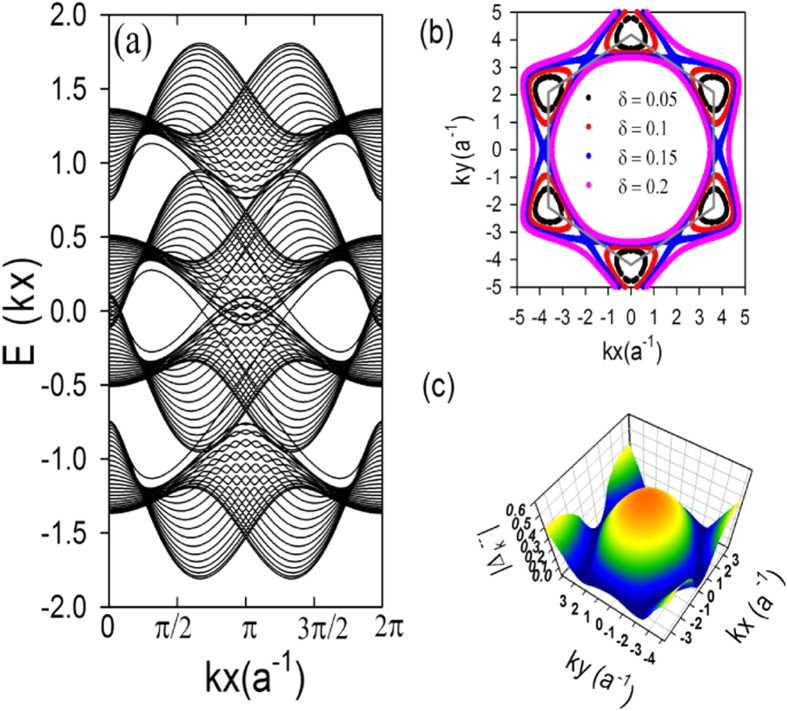
(**a**) The Bogoliubov dispersion *E*(*k*_*x*_) (in unit of *t*) of doped KM-tJ model on a zigzag ribbon with *N* = 56 for *J*/*t* = 0.1, *t*_*SO*_/*t* = 0.8, and *δ* = 0.05. (**b**) The spin-up Fermi surfaces in the normal state of the Kane-Mele model on 2D periodic lattice for *t*_*SO*_/*t* = 1 at various dopings. The spin-down Fermi surfaces are obtained via *K*_+_ → *K*_−_. (**c**) The 3D density plot for the effective intra-band superconducting gap function 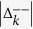 of the Kane-Mele model on 2D periodic lattice (see text and Supplementary materials).

On a finite-sized zigzag ribbon and at a generic doping, the Bogoliubov quasi-particle dispersion shows four doubly-degenerate bulk bands (due to the *S*_*z*_ symmetry of our Hamiltonian) grouped in two pairs (see Fig. 2(a)); it satisfies the particle-hole symmetry with 2*π* periodicity. At low dopings, the normal state Fermi surfaces enclose the Dirac points 
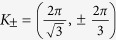
 (see Fig. 2(b)); the d + *id*′ pairing strength is weak near *K*_^38^.

**Figure 3:**
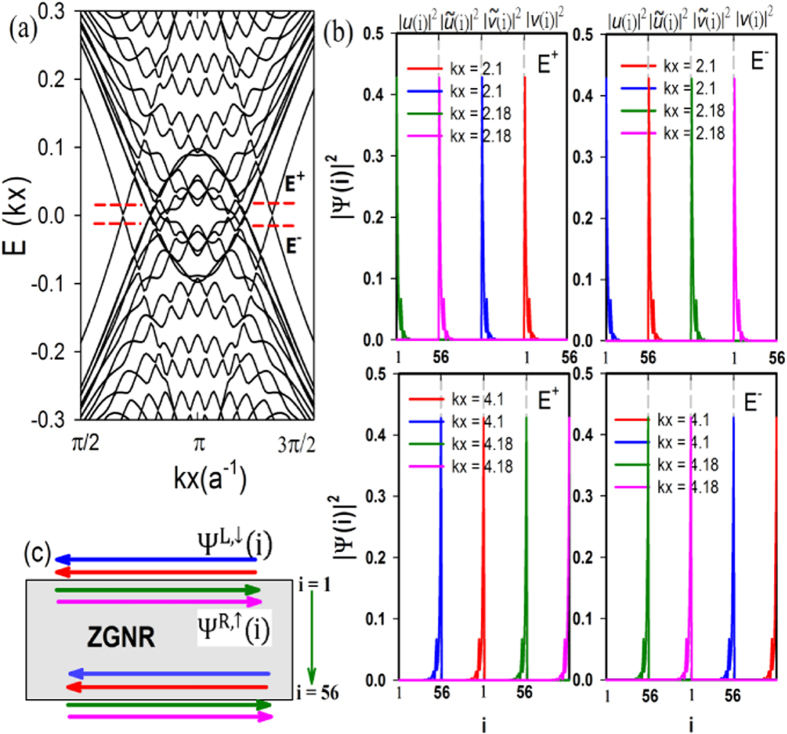
(**a**) Bogoliubov excitation spectrum of Fig. 2(a) near zero energy. (**b**) In the basis of 
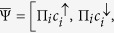

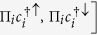
 with *i* = 1 … *N*, the square magnitudes of two pairs of degenerate eigenstate wave-functions associated with the same 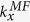 is defined as 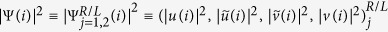 (see text) for a fixed eigenenergy 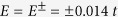 with *i* running (left to right) from *i* = 1 (top edge) to *i* = 56 (bottom edge), corresponding to the helical Majorana fermions. They exhibit an exponential decay from both edges into the bulk. Here, *R*/L refers to the right/left moving state, and 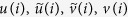 are the corresponding matrix elements. Physical parameters are the same as in (**a**). (**c**) Schematic plot of the helical edge states in (**b**) for *E* = *E*^+^ where same color in (**b**,**c**) refers to the same state. Here, ZGNR refers to the Kane-Mele zigzag nano-ribbon.

Surprisingly, in the regime of a strong SO coupling and weak pairing (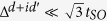), we find the low energy excitations of our model support helical MFs at edges instead of chiral edge states as expected for a chiral *d*–wave superconductor. On a finite-sized zigzag ribbon, we find two Dirac-dispersed lines intersecting at momenta 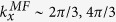 where the Bogoliubov quasi-particle excitation energy vanishes, 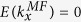 (see Figs 2(a) and 3(a)). Note that for *t*_*SO*_ ≫ Δ_0_, we find bulk gap closes near *k*_*x*_ = 0, *π* in the pseudo-spin-Chern phase (see Fig. 2). This comes as spin-orbital gap of the pure Kane-Mele ribbon gets smaller near Γ point. Upon doping, the P-H symmetry of the bands is imposed, leading to the overlap between particle and hole bands near *k*_*x*_ = 0, *π* for large *t*_*SO*_/Δ_0_. We have checked numerically that all the states near *k*_*x*_ = 0, *π* are indeed bulk states. Nevertheless, when *t*_*SO*_ is of the same order of magnitude as Δ_0_, we find the bulk gap coloses only at the pseudo-spin-Chern-to-chiral phase transition (see Supplementary materials). Near each of these gapless points, two pairs of two-fold degenerate states are generated via intersecting the two Dirac lines by a constant energy at two momentum points 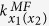, denoted as: 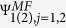 with the subscript 1(2) being the label of the eigenstate at 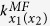 and *j* = 1, 2 the label of two-degenerate eigenstates at a fixed momentum *k*. These four degenerate states are located at the same edge. However, 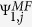 and 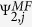 are counter-propagating, while 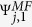 and 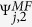 are co-propagating (see Fig. 3(b)).

These features are clearly different from the co-propagating chiral edge states realized either in the chiral *d*-wave superconductivity in doped graphene or by proximity of a *s*–wave superconductor to a quantum anamolous Hall insulators^43^. Instead, the edge states we find fit well to the helical MFs described by the linearly-dispersed Hamiltonian defined by the Bogoliubov quasi-particle operators 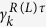 as:


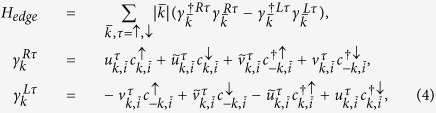


where 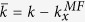, 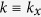, 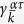 with *α* = *L*, *R* refers to the Bogoliubov quasi-particle destruction operator defined by the coherence factors 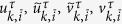, corresponding to the right-moving quasi-particle with “pseudo-spin” *τ* = ↑(↓), and the summation over repeated site index 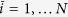 is implied; similarly for 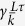. The pair of the degenerate wavefunctions 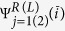 at 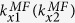 can be expressed as 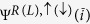, formed by the coherence factors: 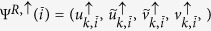, 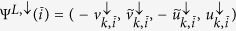; similarly for the other doublet 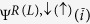. It is clear from Fig. 3(b) that the edge states 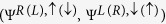 (as well as the Bogoliubov operators 
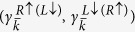
) form pairs (see pink (blue) curve in Fig. 3(b) for 
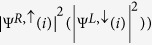
. Furthermore, these Bogoliubov operators with linear dispersion satisfy 
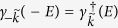
 with 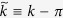 via PH symmetry (see top and bottom panels of Fig. 3(b)). Hence, they can be regarded as examples of helical MFs at edges^15^; the MF zero-modes occur at 
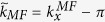
 where 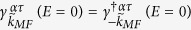. An additional symmetry is observed due to combined P-H and sublattice symmetries: 
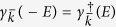
 (see Fig. 3(b)). Our seemingly unexpected results have roots in the competition between TRS SO coupling and TRS breaking chiral *d*–wave superconductivity. It seems to suggest that the TRS protected *Z*_2_ QSH insulating phase of the pure un-doped Kane-Mele model persists up to a finite doping and a finite pairing gap with chiral d-wave narure.

To gain more understanding of this surprising result, we try to identify the non-trivial topological invariant corresponding to the helical edge states we found. We first decompose our 8 × 8 Hamiltonian matrix *M*_*k*_ in Eq. 3 into two separated 4 × 4 matrices .., 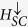 in the new basis 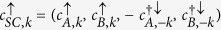, 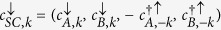, representing the spin-up and spin-down parts of the *M*_*k*_ as^44^:


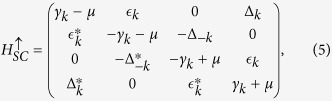


and similarly for 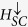. Due to *Sz* and sub-lattice symmetries, two pairs of degenerate bands (one pair with positive and one with negative eigenvalues) are formed in 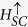 and 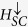. Like the case for the quantum spin-Hall insulator (QSHI), we try to characterize the helical phase of our model in terms of the familar spin Chern number 
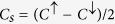
 where 
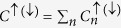
. Here, 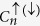 refers to the Chern number of the *n*th filled band in 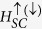: 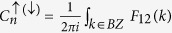 where the integral is done in the first Brillouin zone (BZ), the field strength *F*_12_(*k*) and the associated Berry’s connection *A*(*k*) are defined as: *F*_12_(*k*) ≡ ∂_1_*A*_2_(*k*) − ∂_2_*A*_1_(*k*) and 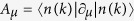 with 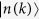 being the normalized eigenvector of the *n*th band in 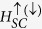
45. However, we find *C*^↑^ = 0 = *C*^↓^ due to the cancellation of *C*_*n*_ within each pair of filled degenerate bands. Therefore, the spin Chern number is zero, *C*_*s*_ = 0.

Nevertheless, 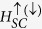 exhibits an additional pseudo-spin symmetry: 
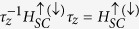
 with *τ*_*z*_ being the *z*–component of the Pauli matrix defined in 4 × 4 matrix within 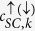 basis, and the pseudo-spin quantum numbers can take ±1. Therefore, the helical phase realized in our system may still be characterized by a different topological number, similar to the spin Chern number, called the pseudo-spin Chern number 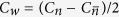 where *n* and 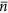 label the two degenerate bands within each 4 × 4 matrix 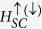, related by 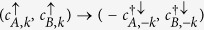, and carry the opposite pseudo-spin quantum number. In the strong SO coupling regime, *t*_*SO*_ ≫ Δ_0_ and for a sizable range of doping around 1/2-filling, we evaluate *C*_*n*_, 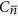 numerically and indeed find 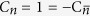, leading to a non-trivial pseudo-spin Chern number *C*_*w*_ = 1 for 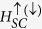. The total pseudo-spin Chern number by summing over contributions from both spin species is therefore 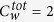 Supplementary materials. Via the bulk-edge correspondence, two pairs of counter-propagating (helical) edge modes are therefore expected, which explains the helical edge states we find numerically via the renormalized mean-field theory. Note that the helical MFs have been known to exist in *Z*_2_ TRITOPs and are protected by the time-reversal symmetry (The existence of two pairs of helical edge states via mean-field analysis agrees perfectly with the total spin Chern number being ±2 via summing over *N*_*W*_ for all four filled bands)^1^,^15^,^46^. However, we provide an example of different kind of helical Majorana edge states here which is not protected by TRS, but by the pseudo-spin symmetry. We may call them quasi-helical edge states to be distinguished from the TRS protected helical edge states. These quasi-helical edge states are robust against disorder or spin-nonconserving interactions, similar to the spin-Chern phase in the QSHIs in the absence of TRS^47^,^48^,^49^.

Deep in the pseudo-spin-Chern phase, our system is well approximated by the effective spin-singlet *p*_*x*_ ± *ip*_*y*_ superconductivity near the two Dirac points *K*_±_. This can be seen when re-expressing the superconducting pairings in terms of the electron operators *ψ*_±,*k*_ which diagonaliz the tight-binding KM Hamiltonian^38^ Supplementary materials. In this basis, the intra-band pairing 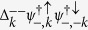 dominates at ground state (see Fig. 2(c) and Supplementary materials). Near *K*_±_ points with *q*_±_ = *K*_±_ + (±*q*_*x*_ + *q*_*y*_), we find 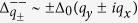, resembling the case of a TRITOPs. In the opposite limit for *t*_*SO*_ ≪ Δ_0_ or sufficiently large doping where the chiral *d*-wave pairing dominates, however, we recover the chiral superconductivity: *N*_*W*_ = 0 and 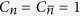, equivalent to the case of doped graphene^35^,^37^.

**Figure 4:**
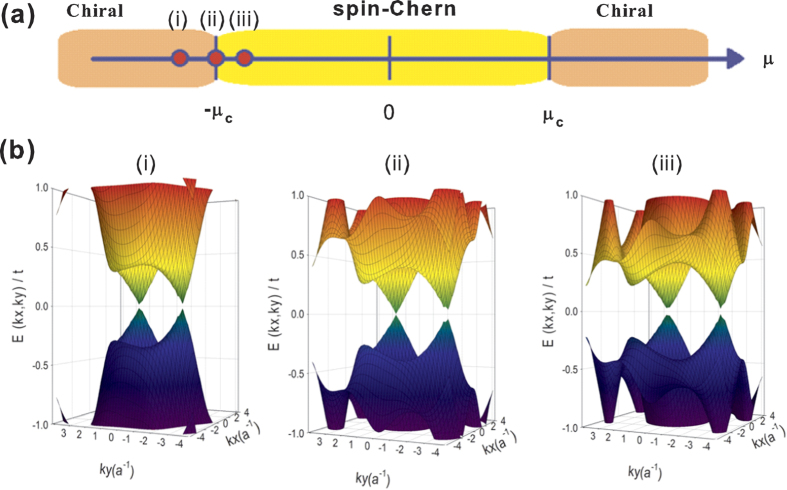
(**a**) Topological phase diagram of doped Kane-Mele t-J model on 2D periodic honeycomb lattice as a function of *μ* for fixed Δ_0_/*t* = 0.3, *t*_*SO*_/*t* = 0.9. Here, we set *χ* = 0, *g*_*t*_ = *g*_*s*_ = 1 for simplicity, and 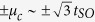 refers to the critical chemical potential at the pseudo-spin-Chern-chiral phase transition. The values of Δ_0_ and *μ* are tuned in a non-self-consistent way. (**b**) Energy dispersion of the two bulk bands close to zero energy. The bulk band gap closes at the phase boundary *μ* ∼ *μ*_c_ at one Dirac point (case (ii) with *μ*/*t* = −1.409, *δ* = 0.43), while it remains finite on either of the two phases (cases (i) with *μ*/*t* = −1.8, *δ* = 0.58 and case (iii) with *μ*/*t* = −1.2, *δ* = 0.34).

A novel pseudo-spin-Chern-to-chiral topological quantum phase transition is identified as Δ_0_/*t*_*SO*_ or *μ*/*t*_*SO*_ is varied (see Supplementary materials)^44^. The generic phase diagram by tuning *μ* (in a non-self-consistent way) at a fixed Δ_0_/*t*_*SO*_ is shown in Fig. 4. For Δ_0_/*t* ≪ 1, the chiral-to-pseudo-spin-Chern phase transition occurs near 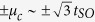 (see Fig. 4(a)). As shown in Fig. 4(b), the bulk band gap closes at the phase boundary *μ* ∼ −*μ*_*c*_ at one Dirac point (case (ii)), while it remains finite on either of the two phases (cases (i) and (iii)).

For Δ_0_ < *t*_*SO*_, we find the critical values of *μ* being at 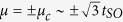. The bulk band gap closes only at the phase transition, while it remains open in either phase^44^,^50^ (see also Supplementary materials). Similar persistence of spin-Chern phase in a TRS breaking magnetic field has been observed experimentally in ref. 51. The pseudo-spin-Chern phase of our system belongs to class *D* topological superconductors, distinct from the TRITOPs^19^,^52^.

## Discussions and Conclusions

Before we conclude, the applicability of our model for the adatom doped graphene and other correlated materials deserves some discussions here. The authors in ref. 26 showed via density functional theory that depending on the elements, adatoms favor either the high-symmetry bridge (B)(center of a bond connecting two carbon atoms), hallow (H) (center of the honeycomb) or the top (T) (on top of a carbon site) positions on the graphen sheet upon doping. In particular, they showed that the hallow (H) position is favored for indium or thallium, which generates an effective intrinsic spin-orbit (SO) coupling of precisely the Kane-Mele type with a sizable enhanced SO coupling (∼20 *meV*) compared to the un-doped graphene. The Kane-Mele model can in principle be realized when adatoms (indium or thallium) are regularly doped on a graphene sheet. Note that the lattice symmetry of graphene is not broken if adatoms are uniformly doped on the H-sites. Meanwhile, the strength of on-site Coulomb interaction *U* in graphene has been estimated via first-principle calculations to be *U*/*t* ∼ 3.3^42^,^53^, which cannot be viewed as a weak coupling or perturbation. Note that in general, a long-range Coulomb repulsion is also present in the un-doped graphene. However, at finite doping, the long-range Coulomb tail in graphene is further suppressed. Since we are interested in the superconducting phase at a finite doping, we consider here only the on-site Coulomb interaction *U* term^42^. The second-order perturbation in *t*/*U* of the Hubbard (with hoping *t* and on-site *U* terms) model leads to our *t*-*J* model with RVB-type antiferromagnetic spin-exchange coupling *J* ∼ *t*. This value of *J*/*t* falls into (intermediate) correlated regime. Though the value of *J*/*t* in graphene may not be large enough to warrant a strong-coupling approach, an effective t-J model of the same form can be derived alternatively by phenomenologically introducing an effective RVB *J* term in the intermediate coupling regime *J* ∼ *t* for graphene where the hoping *t* is not renormalized^36^,^54^. Furthermore, besides graphene, *In*_3_*Cu*_2_*VO*_9_ and *β* − *Cu*_2_*V*_2_*O*_7_ 28 have been proposed recently to be well-described by the *t* − *J* model on honeycomb lattice, while *In*_3_*Cu*_2_*VO*_9_^27^, *β* − *Cu*_2_*V*_2_*O*_7_^29^, *SrPtAs*^55^, *MoS*_2_^56^, and silicene^36^ have been proposed to be chiral *d*-wave superconductors near half-filling. At a general level, we treat our *t* − *J* model within RMFT where the hoping *t* and spin-exchange *J* get renormalized.

In summary, in contrast to the extensively studied chiral (helical) Majorana fermions in *spin-triplet p*_*x*_ + *ip*_*y*_ (*p*_*x*_ ± *ip*_*y*_) superconductivity by applying a magnetic field and/or by proximity effect, we demonstrate for the first time a 2D *spin-singlet* topological superconductor with non-trivial pseudo-spin Chern number in doped correlated Kane-Mele model. Our generic system supports helical counter-propagating Majorana zero modes despite the *d* + *id*′ superconducting pairing gap breaks TRS. This seemingly unexpected feature comes as a result of persistence of spin-Chern phase of the pure Kane-Mele model in the superconducting state upon doping. As *T* → 0, distinct differential conductance spectrum for each pair of Majorana zero mode through differential Andreev conductance in the normal-metal/superconducting (NS) junction is expected^35^,^37^. Further theoretical and experimental investigations are necessary to clarify and realize the exotic helical MFs in doped QSH insulators.

## Methods

Our calculations are based on Renormalized Mean-Field Thoery (RMFT)^33^,^34^. This approach is based on the Gutzwiller projected single-occupancy constraint in the large *U* (onsite Coulomb replusion) limit of the Hubbard model due to the proximity of the Mott insulating ground states. In this limit, the Hubbard model reduces to the *t*-*J* model. The RMFT approach has been known to describe the ground state of *d*–wave cuprate superconductors in qualitative agreement with results via variational Monte Carlo approach^33^,^34^. Projecting out the double occupancy of the *t*-*J* model, the hopping *t* term effectively acquires a reduction factor *g*_*t*_: *t* → *tg*_*t*_ with *g*_*t*_ = 2*δ*/(1 + *δ*), while the spin-exchange *J* term gets enhances by a factor *g*_*s*_: *J* → *g*_*s*_*J* with *g*_*s*_ = 4/(1 + *δ*)^2^. In general, *t*_*SO*_ term gets renormalized as 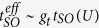, which vanishes at half-filling. However, *t*_*SO*_(*U*) is expected to be strongly enahanced with increasing *U*^23^. For simplicity, at a finite doping, we shall approximately treat 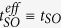 as a constant parameter (The dependence of *t*_*SO*_(*U*) on *U* is rather complictaed. The precise form of *t*_*SO*_(*U*) shall be addressed as a separate issue and it does not affect the qualitative results of the present work). We consider our doped Kane-Mele model in the correlated regime described by the *t*-*J* model. Therefore, the RMFT is an appropriate approach for this purpose. The spin-exchange *J* term is decomposed into the mean-field variables for the superconducting gap function 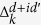 and for the particle-hole excitations *χ*. We numerically solve these mean-field variables self-consistently on a finite-sized zigzag ribbon (*N*_*s*_ = 28 zigzag chains) on honeycomb lattice subject to the chemical potential *μ* and a doping *δ*.

## Additional Information

**How to cite this article**: Sun, S.-J. *et al*. Helical Majorana fermions in 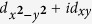-wave topological superconductivity of doped correlated quantum spin Hall insulators. *Sci. Rep*. **6**, 24102; doi: 10.1038/srep24102 (2016).

## Supplementary Material

Supplementary Information
