# Supplementary material for: Helical Majorana fermions in dx2-y2 + idxy-wave topological superconductivity of doped correlated quantum spin Hall insulators
Source: Sci Rep. 2016 Apr 11;6:24102. doi: 10.1038/srep24102 (PMC4827071; doi:10.1038/srep24102)
Supplement: Supplementary Information [file srep24102-s1.pdf]

Supplementary Materials for Helical Majorana fermions in $d_{x^2-y^2} + id_{xy}$-wave topological superconductivity of doped correlated quantum spin Hall insulators

Shih-Jye Sun[1], Chung-Hou Chung[2,3], Yung-Yeh Chang[2], Wei-Feng Tsai[4], and Fu-Chun Zhang[5,6]

[1]*Department of Applied Physics, National University of Kaohsiung, Kaohsiung, Taiwan, R.O.C.*

[2]*Electrophysics Department, National Chiao-Tung University, HsinChu, Taiwan, 300, R.O.C.*

[3]*Physics Division, National Center for Theoretical Sciences, HsinChu, Taiwan, 300 R.O.C.*

[4]*Department of Physics, National Sun Yat-Sen University, Kaohsiung, Taiwan, R.O.C.*

[5]*Department of Physics, Zhejiang University, Hangzhou, China*

[6] *Collaborative Innovation Center of Advanced Microstructures, Nanjing, China*

(Dated: February 18, 2016)

Abstract

In the Supplementary Materials, we provide some details of the calculations mentioned in the main text.

I. THE HAMILTONIAN AND THE MEAN-FIELD SOLUTIONS OF THE KANE-MELE T-J MODEL ON A ZIGZAG RIBBON

Here, we provide in details the Hamiltonian of the Kane-Mele t-J model on zigzag ribbon and the numerical solutions via RMFT. The Hamiltonian matrix is $4N \times 4N$ in size with $N/2$ being the number of zigzag chains along x-axis. Each zigzag chain passes through either A sites or B sites. The ket state of the Hamiltonian is

$$|\Psi\rangle = (c^+_{1\uparrow}c^+_{2\uparrow}\cdots c^+_{N\uparrow}c^+_{1\downarrow}c^+_{2\downarrow}\cdots c^+_{N\downarrow}c_{1\uparrow}c_{2\uparrow}\cdots c_{N\uparrow}c_{1\downarrow}c_{2\downarrow}\cdots c_{N\downarrow})^T \tag{I.1}$$

The Hamiltonian comprises three terms

$$H = H_0 + H_{so} + H_\Delta. \tag{I.2}$$

Here, H_0 represents the nearest-neighbor hopping terms in the matrix form of $(\cdots c^+_{i\sigma}\cdots)H(\cdots c_{j\sigma}\cdots)^T$, given by:

$$\begin{aligned}
&\text{for } i = 1,3,5\cdots N-1,\\
&\qquad H_{0,\sigma\sigma}(i,i+1) = -2tcos(\frac{k}{2}),\\
&\qquad H_{0,\sigma\sigma}(i+1,i) = H_{0,\sigma\sigma}(i,i+1)\\
&\text{for } i = 2,4,6\cdots N,\\
&\qquad H_{0,\sigma\sigma}(i,i+1) = -t,\\
&\qquad H_{0,\sigma\sigma}(i+1,i) = H_{0,\sigma\sigma}(i,i+1),
\end{aligned} \tag{I.3}$$

where $\sigma = \uparrow,\downarrow$ ($+$ or $-$), and $k = k_x$. The matrix elements of the form $(\cdots c_{i\sigma}\cdots)H(\cdots c^+_{j\sigma}\cdots)^T$ is related to $(\cdots c^+_{i\sigma}\cdots)H(\cdots c_{j\sigma}\cdots)^T$ by a minus sign. The H_{so} describes the intrinsic spin-orbit couping with the following matrix form:

for $i = 3, 5, 7 \cdots N-3$

$$
\begin{aligned}
&H_{so,\sigma\sigma}(i, i-2) = \sigma\frac{2}{3}t_{so}sin(\frac{k}{2}),\\
&H_{so,\sigma\sigma}(i-2, i) = H_{so,\sigma\sigma}(i, i-2),\\
&H_{so,\sigma\sigma}(i, i+2) = \sigma\frac{2}{3}t_{so}sin(\frac{k}{2}),\\
&H_{so,\sigma\sigma}(i+2, i) = H_{so,\sigma\sigma}(i, i-2),\\
&H_{so,\sigma\sigma}(i, i) = -\sigma\frac{2}{3}t_{so}sin(k);
\end{aligned}
$$

for $i = 4, 6, 8 \cdots N-2$

$$
\begin{aligned}
&H_{so,\sigma\sigma}(i, i-2) = -\sigma\frac{2}{3}t_{so}sin(\frac{k}{2}),\\
&H_{so,\sigma\sigma}(i-2, i) = H_{so,\sigma\sigma}(i, i-2),\\
&H_{so,\sigma\sigma}(i, i+2) = -\sigma\frac{2}{3}t_{so}sin(\frac{k}{2}),\\
&H_{so,\sigma\sigma}(i+2, i) = H_{so,\sigma\sigma}(i, i-2),\\
&H_{so,\sigma\sigma}(i, i) = \sigma\frac{2}{3}t_{so}sin(k);
\end{aligned}
$$

and the other matrix elements read

$$
\begin{aligned}
&H_{so,\sigma\sigma}(1, 1) = -\sigma\frac{2}{3}t_{so}sin(k),\\
&H_{so,\sigma\sigma}(2, 2) = \sigma\frac{2}{3}t_{so}sin(k),\\
&H_{so,\sigma\sigma}(N-1, N-1) = -\sigma\frac{2}{3}t_{so}sin(k),\\
&H_{so,\sigma\sigma}(N, N) = \sigma\frac{2}{3}t_{so}sin(k),
\end{aligned}
\tag{I.4}
$$

where the matrix elements with the abnormal order is minus the normal order elements. The H_{Δ} is the superconducting pairing gap with the matrix elements of the form

$(\cdots c_{i,\sigma}\cdots)H(\cdots c_{j,-\sigma})^T$ or $(\cdots c^+_{i,\sigma}\cdots)H(\cdots c^+_{j,-\sigma})^T$, given by:

for $i = 1, 3, 5\cdots N-1$

$$H_\Delta(i, i-1+3N) = \Delta_0$$
$$H_\Delta(i-1, i+3N) = \Delta_0$$
$$H_\Delta(i, i+1+3N) = \Delta_1 + \Delta_2$$
$$H_\Delta(i+1, i+3N) = \Delta_1 + \Delta_2$$

similarly

$$H_\Delta(i+N, i-1+2N) = -\Delta_0$$
$$H_\Delta(i-1+N, i+2N) = -\Delta_0$$
$$H_\Delta(i+N, i+1+2N) = -\Delta_1 - \Delta_2$$
$$H_\Delta(i+1+N, i+2N) = -\Delta_1 - \Delta_2$$
$$H_\Delta(i+2N, i-1+N) = H_\Delta(i+N, i-1+2N)^*$$
$$H_\Delta(i-1+2N, i+N) = H_\Delta(i-1+N, i+2N)^*$$
$$H_\Delta(i+2N, i+1+N) = H_\Delta(i+N, i+1+2N)^*$$
$$H_\Delta(i+1+2N, i+N) = H_\Delta(i+1+N, i+2N)^*$$
$$H_\Delta(i+3N, i-1) = H_\Delta(i, i-1+3N)^*$$
$$H_\Delta(i-1+3N, i) = H_\Delta(i-1, i+3N)^*$$
$$H_\Delta(i+3N, i+1) = H_\Delta(i, i+1+3N)^*$$
$$H_\Delta(i+1+3N, i) = H_\Delta(i+1, i+3N)^*,$$

and the other matrix elements read

$$H_\Delta(1, 2+3N) = \Delta_1 + \Delta_2$$
$$H_\Delta(2, 1+3N) = H_\Delta(1, 2+3N)$$
$$H_\Delta(1+N, 2+2N) = -H_\Delta(1, 2+3N)$$
$$H_\Delta(2+N, 1+2N) = -H_\Delta(2, 1+3N)$$
$$H_\Delta(1+2N, 2+N) = H_\Delta(1+N, 2+2N)^*$$
$$H_\Delta(2+2N, 1+N) = H_\Delta(2+N, 1+2N)^*$$
$$H_\Delta(1+3N, 2) = H_\Delta(1, 2+3N)^*$$
$$H_\Delta(2+3N, 1) = H_\Delta(2, 1+3N)^*$$

(I.5)

where Δ_0 is the pairing order $\langle c_{i,\sigma} c_{j,-\sigma} \rangle$, Δ_0, Δ_1 and Δ_2 correspond to the pairing order parameters carrying different phases $0, \frac{2\pi}{3}$ and $\frac{4\pi}{3}$ in $d + id'$-wave superconducting states, respectively.

The mean-field variables are solved self- consistently by minimizing the free energy both for a periodic lattice and a finite-sized ribbon. On a 2D periodic lattice, the results as a function of doping are shown in Fig. 1 (a) and (b). The $d + id'$-wave pairing has the lower free energy compared to that for the extended $s-$wave pairing for the dopings of our interest: $0 < \delta < 0.3$.

II. ADDITIONAL RESULTS OF THE KANE-MELE T-J MODEL ON A ZIGZAG RIBBON

Here, we provide additional results based on our numerical calculations on the doped Kane-Mele $t - J$ model via RMFT.

A. Superconducting states at the edges

In the Bogoliubov quasi-particle spectrum on the zigzag ribbon (see Fig. 2 in the main text), we find two linear-dispersed spectrum crossed at $k_x \sim \pi$ with the energy $E = \pm E_0$ ($E_0 > 0$). They are closely related to the edge states of the pure Kane-Mele model. The negative energy state ($E < 0$) corresponds to the superconducting state at the edges as it supports finite values in superconducting order parameter Δ_0 at the edges and their corresponding eigen-vectors are distributed mainly at the edges (see Fig. 2 (b)).

To investigate further the position dependence of the superconducting gap $\Delta^{d+id'}$, we generalize our mean-field calculations to allow for spatially varying superconducting gap $\Delta(i)$ (instead of the gap with an uniform magnitude). As shown in Fig. 2 (a), the magnitude of gap is enhanced at edges and stay at a constant value in the bulk. This can be understood as the electronic density of states (DOS) of the un-doped pure KM ribbon is enhanced at the edges due to the presence of the helical edge states. As a result, the superconducting pairing strength is enhanced at edges. However, the inhomogeneity of superconducting gap at the edges concerns only with the bulk states at a sizable negative energy. Since we focus in this work on the Majorana fermions near zero energy, far away from the superconducting states at

edges, we therefore ignore this spatial inhomogeneity and consider only the superconducting gap with an uniform magnitude within our RMFT approach.

B. Doping evolution of the Bogoliubov excitation spectrum

Here, we provide the doping evolution of the Bogoliubov excitation spectrum as shown in Fig. 3. The spin-Chern phase with helical Majorana modes survive at low dopings. For $J/t = 0.1, t_{SO}/t = 0.5$, it survives for $0 < \delta < 0.2$. Above $\delta = 0.2$, the superconducting gap vanishes and the system is in the normal state. On the other hand, the chiral phase will prevail in the opposite regime $t_{SO} \ll \Delta_0$ for low dopings. However, the chiral superconductivity has been addressed extensively (see Refs. 28, 36 in the main text). We will leave this issue out of the focus of our present work.

III. TOPOLOGICAL INVARIANT

In this section, we provide details on obtaining the Z_2 topological number (or the pseudo-spin Chern number) in the doped Kane-Mele t-J model following the approach in Ref. 2. First, the TKNN number, a topological (Chern) number, for the $n-$th band is defined as [1]:

$$C_n = \frac{1}{2\pi i} \int_{BZ} dk^2 \; \nabla_{\mathbf{k}} \times \mathbf{A}(\mathbf{k}), \tag{III.1}$$

where $\mathbf{A}(\mathbf{k}) \equiv \langle n(\mathbf{k}) | \nabla_{\mathbf{k}} | n(\mathbf{k}) \rangle$, $|n(\mathbf{k})\rangle$ is the Bloch state in the n–th band up to a normalization coefficient, which satisfies the Schroedinger equation $H(\mathbf{k})|n(\mathbf{k})\rangle = E_n(\mathbf{k})|n(\mathbf{k})\rangle$. The existence of $\mathbf{A}(\mathbf{k})$ implies that there is a non-trivial magnetic field across the first Brillouin Zone (FBZ). The FBZ of the honeycomb lattice we choose for the integration Eq. (III.1) is illustrated in Fig. 4, the parallelogram (area enclosed by red lines) spanned by vectors $\mathbf{q}_1$ and $\mathbf{q}_2$ in momentum space.

In order to numerically calculate the TKNN number covered by the entire FBZ (area enclosed by the solid red lines) in Fig. 4, we first discretize it into $N - 1 \times N - 1$ small flakes of parallelograms such as the smaller parallelogram $abcd$ (enclosed by solid black lines) in Fig. 4. The position of a discretized lattice point (that is the vertices of the small parallelograms

) can be specified by a vector $\mathbf{k}_{i,j} = (\,k_{i1}\;,\;k_{j2})\,,$ for $(i,j) \in [1\;,\;N]$ with its components

$$k_{i1} = \frac{1}{N-1}\left[(i-1)\times q_{1x} + (j-1)\times q_{2x}\right]\;; \tag{III.2}$$

$$k_{j2} = \frac{1}{N-1}\left[(i-1)\times q_{1y} + (j-1)\times q_{2y}\right], \tag{III.3}$$

where q_{ix} and q_{iy} are the components of the vectors $\mathbf{q}_1$ and $\mathbf{q}_2$ with unit lattice spacing $|\bar{\mathbf{a}}| = 1$, they are given by

$$\mathbf{q}_1 = (q_{1x}\;,\;q_{1y}) = \left(\frac{4\pi}{3}\;,\;0\right)\;; \tag{III.4}$$

$$\mathbf{q}_2 = (q_{2x}\;,\;q_{2y}) = \left(\frac{2\pi}{3}\;,\;\frac{2\sqrt{3}\pi}{3}\right). \tag{III.5}$$

The phase difference $\Delta\theta_1(\mathbf{k}_{ij})$ of $\Psi_n(\mathbf{k}) \equiv \langle \mathbf{k}|n(\mathbf{k})\rangle$ travelling from a lattice point $\mathbf{k}_{i,j}$ to $\mathbf{k}_{i+1,j}$ can be computed to be

$$\begin{aligned}\Delta\theta_1(\mathbf{k}_{ij}) &= -\,i\ln\left[\frac{\Psi_n^\dagger(\mathbf{k}_{i,j})\,\Psi_n(\mathbf{k}_{i+1,j})}{|\Psi_n^\dagger(\mathbf{k}_{i,j})\,\Psi_n(\mathbf{k}_{i+1,j})\,|}\right]. \\ &\equiv -\,i\ln U_1(\mathbf{k}_{ij})\,,\end{aligned} \tag{III.6}$$

where

$$U_1(\mathbf{k}_{i,j}) \equiv \frac{\Psi_n^\dagger(\mathbf{k}_{i,j})\,\Psi_n(\mathbf{k}_{i+1,j})}{|\Psi_n^\dagger(\mathbf{k}_{i,j})\,\Psi_n(\mathbf{k}_{i+1,j})\,|} \tag{III.7}$$

The subscript "1" of $\Delta\theta_1$ and $U_1(\mathbf{k}_{ij})$ represents that Ψ_n travels along the direction of $\mathbf{q}_1$. Sum over $\Delta\theta$ of the four sides of the small parallelogram *abcd* in Fig. 4 can one obtain the total phase shift of the Bloch wavefunction on the n–th band after circling around that small parallelogram. Compare with Eq. (III.1), we define the lattice $U(1)$ gauge field strength F^n_{ij} by

$$F^n_{ij} \equiv \ln U_1(\mathbf{k}_{i,j})\,U_2(\mathbf{k}_{i+1,j})\,U_1^{-1}(\mathbf{k}_{i,j+1})\,U_2^{-1}(\mathbf{k}_{i,j}) \tag{III.8}$$

Finally, we can calculate the TKNN number on the lattice associated the n-th band by

$$C_n = \frac{1}{2\pi i}\sum_{i,j} F^n_{ij}. \tag{III.9}$$

For the same band we can further define a topological number, so-called the pseudo-spin Chern number, as:

$$N_W = \frac{C_n - C_{\bar{n}}}{2}, \tag{III.10}$$

where $C_{\bar{n}}$ is the TKNN number of the other degenerate eigenvector of the same band. Numerically, we obtain $C_{\bar{n}} = -C_n$, thus N_W is either 1 or -1. We checked numerically that for the parameters used in Fig. 3 of the main text $|N_W|$ approaches to 1. The total $|N_W| = 2$ once the two pairs of occupied degenerate bands are included.

In the other extreme limit, $\Delta \gg t_{SO}$, we found that $N_{TKNN} \equiv C_n + C_{\bar{n}} = \pm 2$, and $N_W = 0$, as expected for $d + id'$ superconductivity in doped graphene [3]. Further studies suggest the existence of a quantum phase transition at a critical ratio of Δ/t_{SO} separating the spin-Chern phase with $N_W = \pm 1$ from the chiral (TKNN) phase with $N_{TKNN} = \pm 2$ (see Ref. 3).

IV. EFFECTIVE TRS SUPERCONDUCTIVITY NEAR THE DIRAC POINTS

In this section, we derive the low-energy effective TRS superconducting pairing of our model near Dirac points. We re-express the $d + id'$ superconducting order parameter in terms of the electron operators $\psi_{\pm,k}$ which diagonalizes the tight-binding KM Hamiltonian with the corresponding eigenvalue $E^{\pm} = \pm(\sqrt{\epsilon_k^2 + \gamma_k^2} - \mu)$ (see Ref. 5):

$$\begin{pmatrix} c^{\uparrow}_{A,k} \\ c^{\uparrow}_{B,k} \end{pmatrix} = \begin{pmatrix} -\alpha_k^- & -\alpha_k^+ \\ \beta_k^- & \beta_k^+ \end{pmatrix} \begin{pmatrix} \psi^{\uparrow}_{+,k} \\ \psi^{\uparrow}_{-,k} \end{pmatrix} \tag{IV.1}$$

and

$$\begin{pmatrix} c^{\downarrow}_{A,k} \\ c^{\downarrow}_{B,k} \end{pmatrix} = \begin{pmatrix} \alpha_k^+ & \alpha_k^- \\ \beta_k^+ & \beta_k^- \end{pmatrix} \begin{pmatrix} \psi^{\downarrow}_{+,k} \\ \psi^{\downarrow}_{-,k} \end{pmatrix} \tag{IV.2}$$

where $\alpha_k^{\pm} = \beta^{\pm} \times \frac{\epsilon_k(\gamma_k \pm \sqrt{\epsilon_k^2+\gamma_k^2})}{|\epsilon_k|^2}$, $\beta^{\pm} = \frac{|\epsilon_k|}{\sqrt{|\epsilon_k|^2+(\gamma_k\pm\sqrt{\epsilon_k^2+\gamma_k^2})^2}}$, and $(\alpha_k^{\pm})^* = -\alpha_{-k}^{\mp}$, $\beta^{\pm} = |\alpha_k^{\mp}|$. The $d + id'$ superconducting pairing term can be re-written as:

$$
\begin{aligned}
H_\Delta &= \sum_k \Delta_k^{d+id'}(c_{A,k}^{\dagger\uparrow} c_{B,k}^{\dagger\downarrow} - c_{A,k}^{\dagger\downarrow} c_{B,k}^{\dagger\uparrow}) + h.c. \\
&= \sum_k [\Delta_k^{--} \psi_{-,k}^{\dagger\uparrow} \psi_{-,-k}^{\dagger\downarrow} + \Delta_k^{++} \psi_{+,k}^{\dagger\uparrow} \psi_{+,-k}^{\dagger\downarrow} \\
&+ \Delta_k^{+-} \psi_{+,k}^{\dagger\uparrow} \psi_{-,-k}^{\dagger\downarrow} + \Delta_k^{-+} \psi_{-,-k}^{\dagger\uparrow} \psi_{+,k}^{\dagger\downarrow}] + h.c., \\
\Delta_k^{++(--)} &= |\alpha_k^{+(-)}||\alpha_k^{-(+)}| \\
&\times [\Delta_k^{d+id'} e^{i\theta(\alpha_k^{+(-)})} + \Delta_{-k}^{d+id'} e^{-i\theta(\alpha_k^{+(-)})}], \\
\Delta_k^{+-(-+)} &= \pm|\alpha_k^{+(-)}|^2 e^{i\theta(\alpha_k^{+(-)})} [\Delta_k^{d+id'} - \Delta_{-k}^{d+id'}],
\end{aligned}
\tag{IV.3}
$$

where $\alpha_k^{\pm} = |\alpha_k^{\pm}| e^{i\theta(\alpha_k^{\pm})}$ with $\theta(\alpha_k^{\pm})$ being th ephase of $\alpha_k^{\pm}$. In the presence of a finite SO coupling and hole-doping, the intra-band pairing of the lower band $\psi_{-,k}^{\dagger\uparrow}\psi_{-,-k}^{\dagger\downarrow}$ with pairing gap function Δ_k^{--} dominates. The gap function $\Delta_{q_\pm}^{--}$ near Dirac points $K_\pm = (\frac{2\pi}{3\sqrt{3}}, \pm\frac{2\pi}{3})$ with $q_\pm = K_\pm + (\pm q_x, q_y)$ behaves to leading order in $|q|$ as an effective spin-singlet $p_x \pm ip_y$-like superconductivity:

$$
\Delta_{q_\pm}^{--} \sim -a_0 \Delta_0 i (q_x \mp i q_y) \tag{IV.4}
$$

with $a_0 = \frac{3te^{i\pi/3}}{12\sqrt{3}t_{SO}}$. Hence, our system behaves as an effective TRITOPs. In deriving Eq. (IV.4), we have used the following results:

$$
\begin{aligned}
\epsilon_{q_\pm} &\approx \pm\frac{3t}{2}(q_y + iq_x), \\
\alpha_{q_-}^{-} &\approx -\frac{\epsilon_{q_-}}{2\sqrt{3}t_{SO}}, \\
\alpha_{q_-}^{+} &\approx \epsilon_{q_-}/|\epsilon_{q_-}|.
\end{aligned}
\tag{IV.5}
$$

We have also used the approximated forms of $d + id'$ pairing gap $\Delta^{d+id'}(q)_\pm$ near the Dirac points, shown to be effectively a mixture of $s-$wave like (near K_+) and $p_x + ip_y-$wave like (near K_-) superconducting pairing [4]:

$$
\begin{aligned}
\Delta^{d+id'}(q_+) &\approx 3\Delta_0 e^{i4\pi/3}, \\
\Delta^{d+id'}(q_-) &\approx \frac{3}{2}\Delta_0 e^{i\pi/3}(q_y - iq_x).
\end{aligned}
\tag{IV.6}
$$

[1] Qi, X. L. & Zhang, S. C. Topological insulators and superconductors. *Rev. Mod. Phys.* **83,** 1057-1110 (2011).

[2] Fukui, T., Hatsugai, Y. & Suzuki, H. Chern numbers in discretized Brillouin zone: efficient method of computing (spin) Hall conductances. *J. Phys. Soc. Jpn.* **74,** 1674-1677 (2005).

[3] Huang, S. M., Tsai, W. F., Chung, C. H. & Mou, C. Y. Duality in topological superconductors and topological ferromagnetic insulators in a honeycomb lattice. arXiv:1601.01098 (2016).

[4] Jiang, Y. J., Yao, D. X., Carlson, E., Chen, H. D. & Hu, J. P. Andreev conductance in the $d + id'$-wave superconducting states of graphene. *Phys. Rev. B* **77,** 235420 (2008).

[5] Wu, W., Scherer, M. M., Honerkamp, C. & Le Hur, K. Correlated Dirac particles and superconductivity on the honeycomb lattice. *Phys. Rev. B* **87,** 094521 (2013).

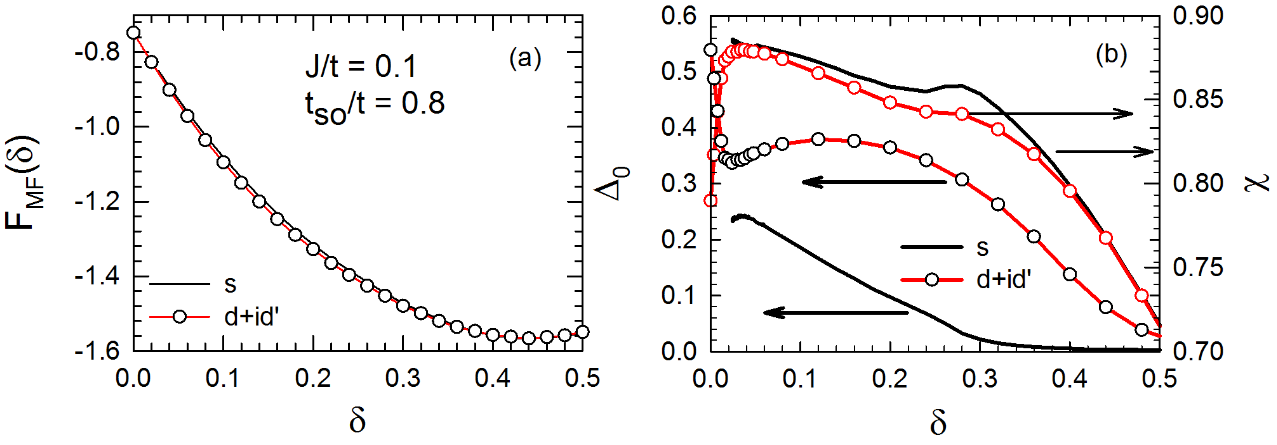

FIG. 1: (Color online) (a). Mean-field free energy F_{MF} versus doping δ of doped Kane-Mele model on 2D periodic lattice for extended s-wave and $d + id'$-wave superconducting pairing symmetries. (a). Mean-field variables Δ_0 (strength of pairing gap) and χ-field versus doping for (b). The parameters used are: $J/t = 0.1$, $t_{SO}/t = 0.8$.

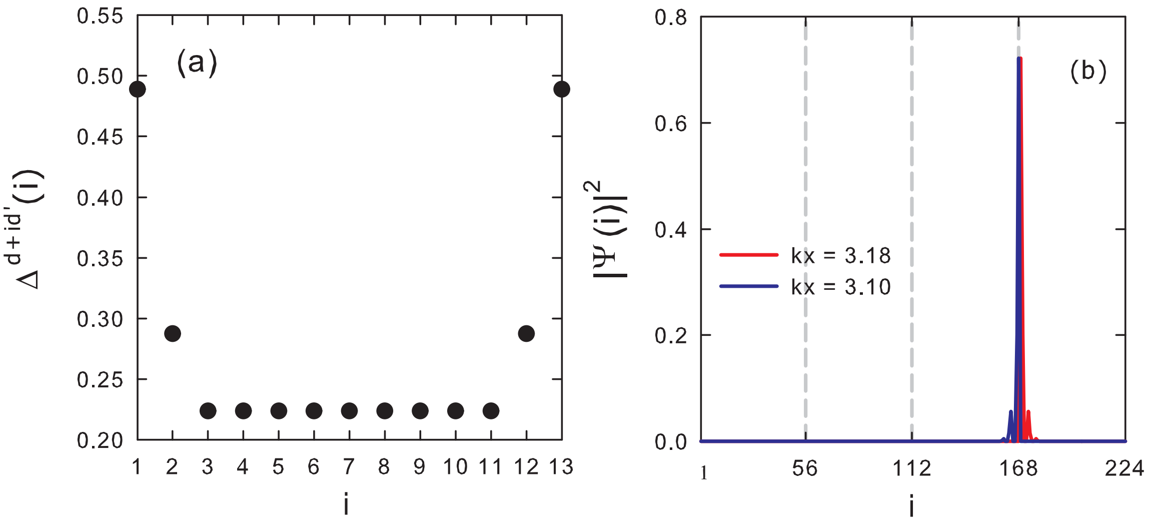

FIG. 2: (Color online) (a) Superconducting gap $\Delta^{d+id'}$ as a function of position in the zigzag ribbon for $N = 26$ (or $N/2 = 13$ zigzag chains). (b) Square magnitude of wave functions $|\Psi(i)|^2$ at the edge in the superconducting states with an energy $E/t = -0.613$ as a function of position $i = 1 \cdots N$ in the zigzag ribbon with $N = 56$. Here, we take the uniform mean-field solution for $\Delta^{d+id'}(i) = 0.22$ for $N = 56$. The parameters used in (a) and (b) are: $J/t = 0.1$, $\delta = 0.15$, $t_{SO}/t = 0.5$.

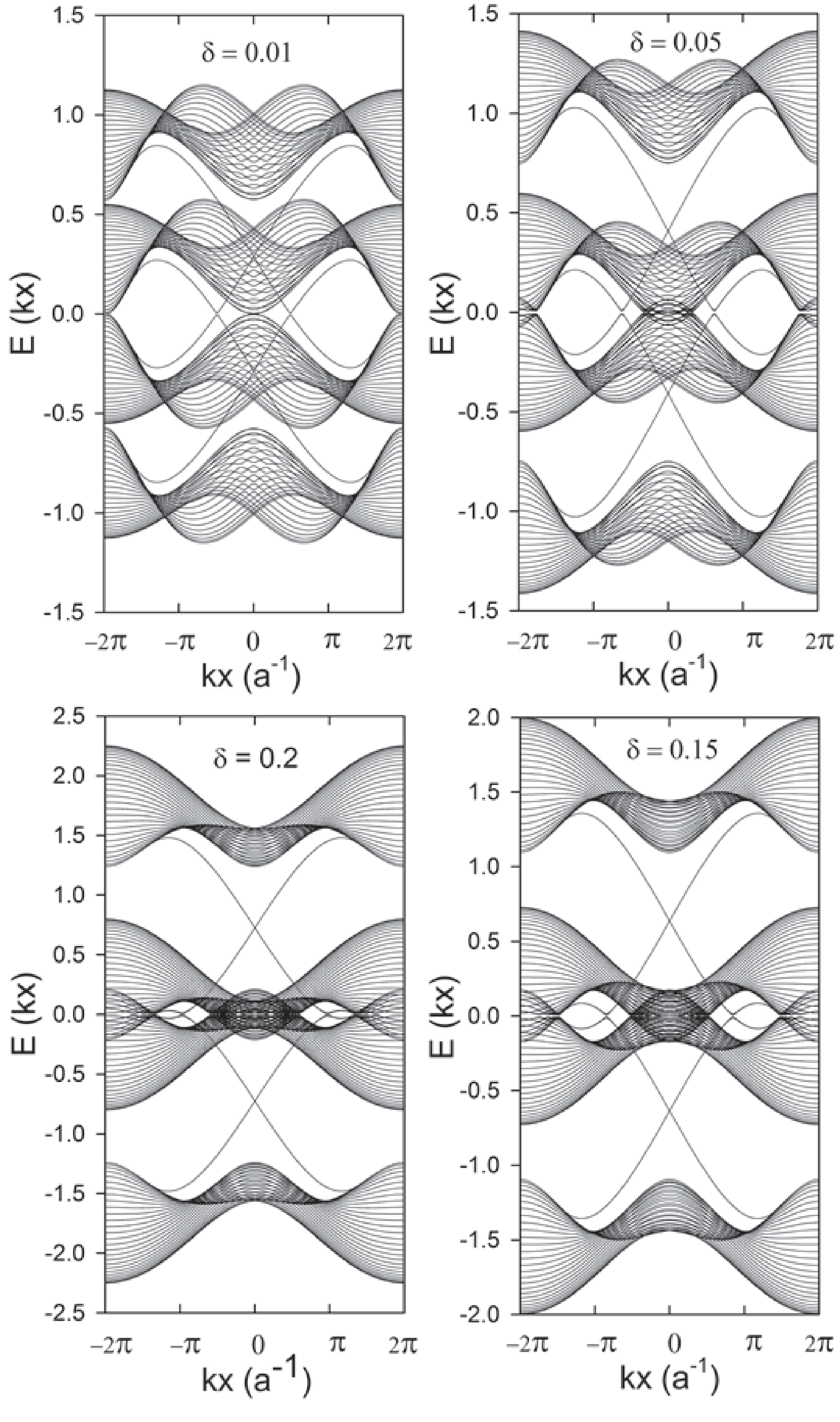

FIG. 3: Bogoliubov excitation spectrum of the Kane-Mele t-J model for different dopings. The parameters used here are: $J/t = 0.1$, $t_{SO}/t = 0.5$.

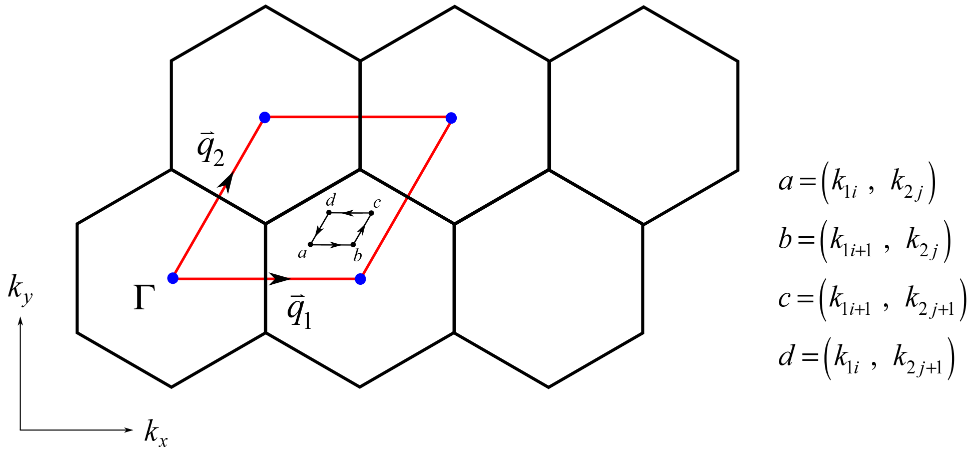

FIG. 4: The parallelogram enclosed by red solid lines is the area of entire FBZ with $\Gamma = (k_x = 0, k_y = 0)$. The small flake of parallelogram $abcd$ is the smallest unit after discretizing the FBZ for numerical calculation.
